# Clinical research on secure multi-party computation technology for auxiliary respiratory pathogen diagnosis and treatment design in preschool-aged children

**DOI:** 10.3389/fped.2026.1798007

**Published:** 2026-08-25

**Authors:** Xi Zhai, Pengqian Zhang

**Affiliations:** Department of Information Center, Hebei Children's Hospital, Shijiazhuang, China

**Keywords:** clinical decision support, data security, immunocompromised, preschool-aged children, respiratory pathogen diagnosis, secure multi-party computation

## Abstract

**Background:**

Privacy-preserving integration of respiratory pathogen, host and treatment data remains difficult in preschool-aged children. We evaluated secure multi-party computation (SMC) as clinician-reviewed decision support for respiratory pathogen diagnosis and treatment planning, while distinguishing technical validation from clinical efficacy.

**Methods:**

This prospective observational study with a parallel in silico comparator was conducted at a tertiary paediatric centre in 2023 among children aged 3–6 years undergoing respiratory pathogen testing and treatment planning. A three-party SMC system using Brakerski-Gentry-Vaikuntanathan homomorphic encryption analysed pathogen markers, clinical features, immune status, inflammatory biomarkers and prespecified treatment rules. Diagnostic-support metrics were calculated against final clinician-documented diagnoses; the non-encrypted comparator assessed encryption overhead and output equivalence.

**Results:**

Among 1,380 enrolled children, *Mycoplasma pneumoniae* immunoglobulin M (IgM), an atypical bacterial marker, had the highest positivity rate (41.30%), followed by influenza B (9.13%) and influenza A (7.39%) virus IgM. The SMC system showed 94.7% diagnostic-support concordance, macro-AUROC 0.962, precision 94.0%, recall 95.1% and F1-score 94.5%. Mean processing time was 2.3 ± 0.5 seconds, uptime was 99.97% and no cryptographic failure or unauthorised access event was detected. Treatment outcomes were interpreted descriptively.

**Conclusions:**

SMC-based clinician-reviewed decision support was feasible for privacy-preserving auxiliary respiratory pathogen diagnosis and treatment planning in preschool-aged children at a single tertiary centre. The findings supported technical feasibility and reference-standard concordance but did not establish superiority over standard physician care. Broader multicentre, age-specific validation is required.

## Introduction

The rapid evolution of respiratory infections and their complex interaction with various pathological conditions have created major challenges in clinical diagnosis and treatment design for preschool-aged children (1). Recent advances in medical informatics have highlighted the potential of secure computational methods to support diagnostic workflows and treatment planning, particularly in cases involving multiple pathogen markers and comorbidities (2). The integration of secure multi-party computation (SMC) into clinical decision support systems has emerged as a promising approach to enhance privacy-preserving respiratory pathogen data processing while maintaining patient data privacy (3).

The complexity of respiratory pathogen diagnosis in children is further compounded by the frequent co-existence of multiple conditions, ranging from oncological disorders to autoimmune diseases (4). Traditional diagnostic approaches often face limitations in processing multidimensional clinical data, potentially leading to delayed or suboptimal treatment decisions (5). Different hospitals and laboratories often face difficulties in sharing sensitive data, such as pathogen genotypes and child test results, which limits source tracing of epidemic spread, mutation monitoring and treatment optimisation (6). The advancement of artificial intelligence (AI) and machine learning techniques in healthcare has demonstrated remarkable potential for pattern recognition and risk stratification. However, concerns regarding data security and privacy have limited their widespread clinical implementation (7).

Secure multi-party computation represents a cryptographic paradigm that enables multiple parties to jointly compute functions over their inputs while keeping those inputs private (8). In the context of respiratory infection management in preschool-aged children, SMC technology offers a framework for integrating diverse diagnostic parameters and treatment protocols while ensuring compliance with stringent medical data protection regulations (9). The limitations of respiratory infection diagnosis lie not only in laboratory technology itself but also in barriers to privacy-preserving data sharing. Secure multi-party computation provides a feasible technical pathway to reduce these barriers and may support diagnostic concordance, response speed and public health decision-making capacity while ensuring privacy.

Children present unique challenges in respiratory pathogen diagnosis and treatment, particularly because of age-specific immune responses and the presence of underlying conditions (10). The increasing prevalence of immunocompromised states in children, especially those undergoing chemotherapy or with primary inborn errors of immunity, necessitates more sophisticated diagnostic and treatment approaches (11). Furthermore, the rising incidence of respiratory infections in children with chronic diseases has emphasised the need for more precise and personalised therapeutic strategies (12).

Contemporary research has demonstrated that integrated computational approaches can support the accuracy and consistency of respiratory pathogen diagnosis and treatment planning (13). However, the implementation of such systems in clinical settings requires careful consideration of both technical capabilities and practical limitations (14). The potential of SMC technology to address these challenges while maintaining data security has been increasingly recognised in the recent medical informatics literature (15).

Despite these advances, a considerable gap remains in understanding the practical feasibility and diagnostic-support performance of SMC technology in real-world clinical settings, particularly in respiratory pathogen diagnosis and treatment design for preschool-aged children (16). Although theoretical frameworks have been well established, empirical evidence regarding clinical workflow integration, implementation costs, operational complexity and staff training requirements remains limited (17). The intersection of respiratory diagnostics, preschool paediatrics and secure computation presents a focused opportunity for innovative research that could support safer privacy-preserving clinical decision support (18).

The purpose of this study was to evaluate the clinical feasibility and reference-standard diagnostic-support performance of an SMC-based system in respiratory pathogen diagnosis and treatment design for preschool-aged children, with a particular focus on cases involving multiple pathogen markers and complex comorbidities.

## Methods

### Study design and setting

This prospective observational study was conducted at a tertiary paediatric medical centre between January 2023 and December 2023. The study protocol was approved by the Ethics Committee of Children's Hospital of Hebei (approval number: YYLSNo.2026047; approval date: July 8th, 2026), and written informed consent was obtained from the legal guardians of all participants. The SMC system operated as auxiliary decision support; it did not autonomously direct care, and all outputs were reviewed by attending physicians before clinical implementation.

### Study Population

Eligible children were aged 3–6 years and underwent respiratory pathogen diagnostic testing and subsequent treatment. The inclusion criteria encompassed children requiring screening for multiple respiratory pathogen markers. Children were excluded if they had incomplete clinical data, had previously participated in other clinical trials or if their guardians withdrew consent during the study period. Children outside the 3–6-year age range were not included in the validation cohort, and the diagnostic thresholds, treatment decision rules and immune-defect safety flags were therefore prespecified for preschool-aged children only.

### Secure multi-party computation system development and implementation

**Figure 1:**
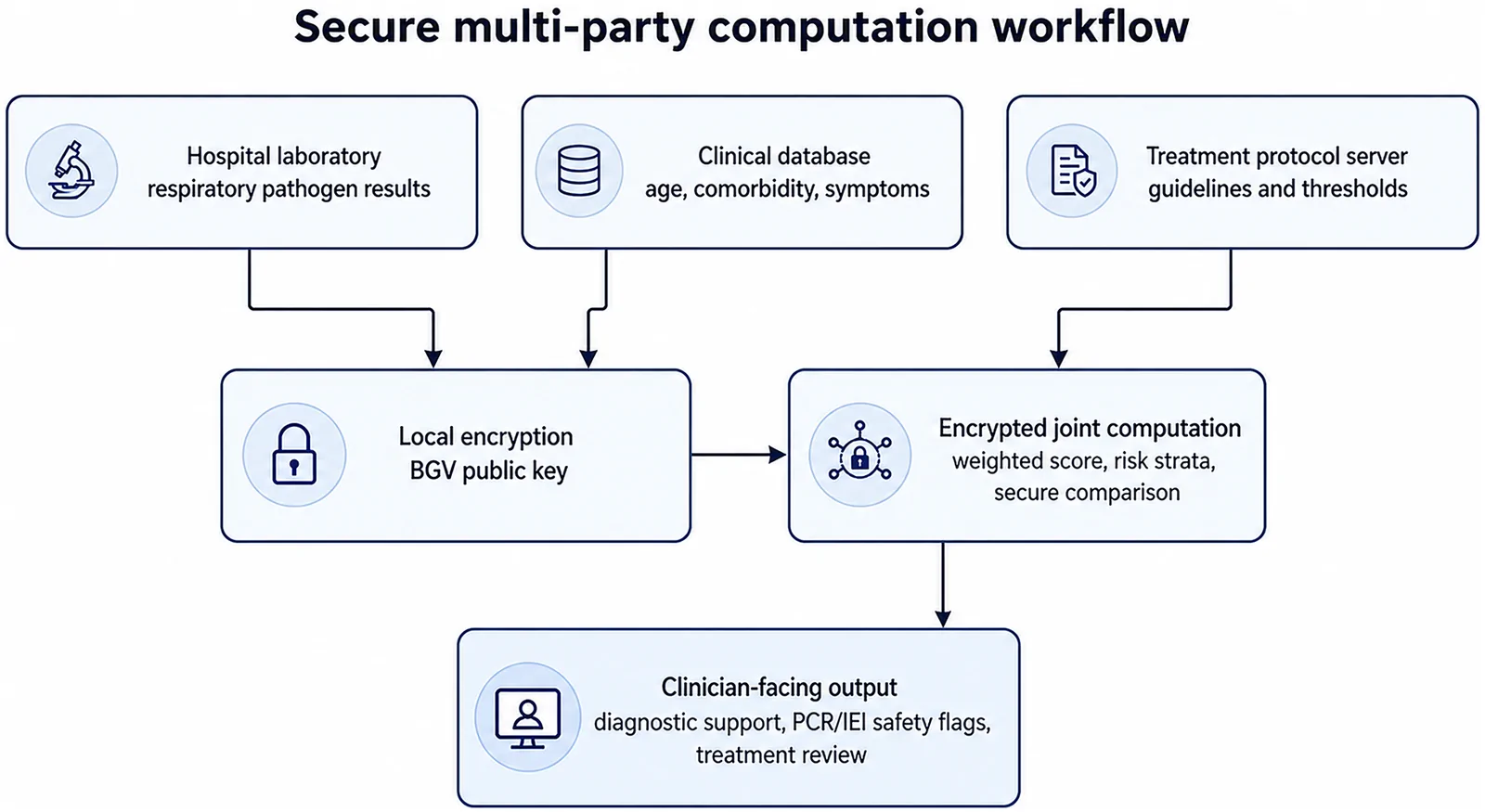
Cryptographic computational workflow of the secure multi-party computation-based respiratory pathogen decision-support system. The figure shows local encryption by three data-holding parties, encrypted joint computation, secure comparison and clinician-facing output with polymerase chain reaction and inborn-error-of-immunity safety flags. Abbreviations: BGV, Brakerski–Gentry–Vaikuntanathan; IEI, inborn error of immunity; PCR, polymerase chain reaction; SMC, secure multi-party computation.

The SMC system was developed using a three-layer architecture with clearly defined data ownership and protection mechanisms. Three parties participated in the secure computation: the hospital laboratory (Party A), which held encrypted respiratory pathogen test results, including atypical bacterial and viral markers; the clinical database (Party B), which maintained encrypted patient demographics, symptoms, immune status and medical history; and the treatment protocol server (Party C), which contained encrypted treatment guidelines, antimicrobial stewardship rules and decision thresholds. Each party encrypted its data locally using public keys before sharing encrypted values for computation. For homomorphic encryption implementation, the Brakerski–Gentry–Vaikuntanathan scheme was used with the following specifications: polynomial modulus degree 16,384; coefficient modulus, 438 bits; plaintext modulus, 65,537; and fixed-point representation with 6-decimal precision, scaled to integers by multiplying by 10^6. The deployed clinical decision circuit was deliberately shallow, with multiplicative depth limited to two because the algorithm used linear weighted scoring followed by low-degree polynomial risk stratification and secure comparison. The weighted score was expressed as E(Score) = ∑i(wi × E[Vi]), where E(Vi) represents an encrypted pathogen or host-feature value. Treatment matching used encrypted decision trees and secure comparison functions, whereas multi-party aggregation used secure addition of encrypted clinical parameters across parties. Public key generation used a distributed key generation protocol; each party held a private-key share, decryption required collaboration between at least two parties, and re-encryption with key rotation was performed every 30 days. The cryptographic computational workflow is illustrated in Figure 1. The clinical deployment was benchmarked on a CPU-based hospital private-cloud server environment with 24 physical CPU cores, 128 GB RAM, NVMe solid-state storage and a 10 Gb/s internal network; no GPU acceleration was used. For institutions implementing the same shallow BGV decision circuit at comparable case volumes, the baseline operational requirement was defined as at least 16 CPU cores, 64 GB RAM, solid-state storage and a 1 Gb/s low-latency intranet connection, together with role-based access control and routine key-management capacity.

### Data collection and security measures

Clinical data were collected through a standardised electronic case report form. The collected parameters included demographic information, respiratory pathogen test results, clinical symptoms, underlying conditions, treatment protocols, outcome measures and, when available, molecular test cycle threshold values, C-reactive protein levels and procalcitonin levels. All data were encrypted at the point of collection using asymmetric encryption protocols. Access to the system was restricted through multi-factor authentication and role-based access control. The feature vector did not treat single-point immunoglobulin M (IgM) results as the sole determinant of acute infection when molecular or inflammatory marker data were available.

Respiratory pathogen testing was performed using standardised immunological assays in the hospital's central laboratory. Results were classified as positive, weakly positive or negative according to established clinical thresholds. *Mycoplasma pneumoniae* and *Chlamydia pneumoniae* were classified as atypical bacteria rather than viruses, and treatment logic for these markers was separated from the viral supportive-care pathway. The system also included a safety flag for children with suspected inborn errors of immunity, particularly humoral immune defects, recurrent severe infections, low immunoglobulin levels or recent immunoglobulin replacement therapy. In such cases, negative IgM serology was not interpreted as excluding infection, and molecular testing, such as polymerase chain reaction (PCR), was recommended.

### Treatment design process

The SMC system utilised secure computation protocols to analyse treatment options based on respiratory pathogen results, patient characteristics and comorbidity profiles. The clinical algorithm was a prespecified rule-based weighted score rather than a model trained on the study outcomes. The normalised diagnostic-support score assigned weights of 0.30 to pathogen-marker category and serological strength, 0.20 to molecular confirmation when PCR or cycle-threshold data were available, 0.15 to respiratory symptom severity and duration, 0.15 to immune status, 0.10 to inflammatory biomarkers including C-reactive protein and procalcitonin and 0.10 to comorbidity, medication contraindication and local resistance or stewardship modifiers. Scores of ≥0.70 triggered pathogen-directed treatment review, scores of 0.40–0.69 triggered additional testing or specialist review and scores of <0.40 supported observation, symptomatic management or repeat assessment. For *M. pneumoniae*, the decision rules distinguished atypical bacterial pneumonia from viral pneumonia, incorporated local macrolide-resistant *M. pneumoniae* risk and prompted antimicrobial stewardship review rather than automatic classification into the supportive-care pathway. The system's recommendations were reviewed by attending physicians before implementation.

For score construction, positive, weakly positive and negative serological markers were encoded as 1.00, 0.50 and 0.00, respectively; PCR-confirmed infection was encoded as 1.00 and PCR-negative status as 0.00 when testing was available. C-reactive protein and procalcitonin were normalised to 0–1 severity bands according to local laboratory thresholds. Safety overrides were applied before final recommendation: suspected inborn errors of immunity, recent immunoglobulin therapy, sepsis, rapidly progressive disease, medication contraindications or conflicting test results forced clinician review and prevented automatic treatment classification.

### Outcome assessment

To evaluate the impact of encryption on system performance, a parallel in silico computational comparison was conducted. The comparator consisted of the same enrolled cases analysed using an identical diagnostic and treatment-support algorithm without SMC encryption. This design was used to assess encryption-related computational overhead and output equivalence; it did not constitute a separate clinical control group and was not intended to demonstrate superiority over current physician-led clinical practice.

The primary technical outcomes included diagnostic-support concordance with the clinical reference standard, AUROC, precision, recall, F1-score, encrypted-computation time, output equivalence between encrypted and non-encrypted execution, system uptime and monitored security events. Clinical treatment outcomes were secondary descriptive outcomes of the whole care pathway and were not modelled as effects caused by the SMC layer. Treatment outcomes were classified as cured, improved, unchanged or deteriorated according to standardised clinical criteria. System performance was evaluated using metrics such as computation time, accuracy of secure calculations, physician concordance, system uptime and maintenance of data security.

Diagnostic-support performance was evaluated against the final clinician-documented diagnosis recorded in the electronic case report form, supported by standard-of-care laboratory evidence, including PCR or molecular testing when available, serology interpreted according to laboratory thresholds, inflammatory biomarkers, clinical course, follow-up findings and discharge diagnosis. True-positive, false-positive, true-negative and false-negative classifications were therefore based on agreement or disagreement between the SMC output and this composite clinical reference standard. These metrics measure reference-standard concordance and should not be interpreted as proof that the SMC system improved patient outcomes compared with standard physician care.

### Statistical analysis

Statistical analyses were performed using encrypted data within the SMC framework and verified against plaintext aggregate outputs generated by the non-encrypted computational comparator. Continuous variables were expressed as means with standard deviations or medians with interquartile ranges, as appropriate. Categorical variables were presented as frequencies and percentages. Secure *χ*2 and Fisher's exact tests were used for categorical comparisons, secure t-tests were used for normally distributed continuous variables and homomorphic comparison functions were used to derive encrypted contingency tables. Diagnostic-support performance was summarised using accuracy, AUROC, precision, recall, F1-score, false-positive rate and false-negative rate against the composite clinical reference standard. For the computational comparison, a two-sided *p*-value <0.05 was considered statistically significant. No statistical comparison with standard physician care was performed because no physician-care control group existed; *p*-values comparing SMC and non-encrypted outputs therefore represent technical equivalence and latency assessment rather than clinical efficacy. With 1,380 cases, the study had greater than 90% statistical power to estimate an expected diagnostic-support concordance of approximately 95% within a two-sided 95% confidence interval width of less than 2.5 percentage points and to detect a 5% difference in paired accuracy between encrypted and non-encrypted outputs. The statistical analysis plan and aggregate outputs were reviewed by a study statistician before database lock.

### System validation and quality control

The SMC system underwent rigorous validation through a three-phase process that included technical verification, reference-standard concordance assessment and security testing. Regular quality-control measures were implemented to ensure system reliability and data integrity. Independent audits of the security protocols were conducted monthly throughout the study period, and statistical outputs were reviewed by a study statistician before database lock.

### Follow-up protocol

Children were followed up according to their clinical conditions, with a minimum follow-up period of 3 months. Follow-up assessments included respiratory pathogen monitoring when clinically indicated, molecular testing when required, clinical evaluation and treatment response assessment. All follow-up data were integrated into the SMC system for continuous analysis and treatment optimisation.

### Ethical considerations and data protection

The study adhered to the principles of the Declaration of Helsinki and followed international guidelines for clinical research involving children. The protocol was approved by the Ethics Committee of Children's Hospital of Hebei (approval number: YYLSNo.2026047; approval date: July 8th, 2026). Data protection measures complied with General Data Protection Regulation and Health Insurance Portability and Accountability Act requirements. All personnel involved in the study completed specialised training in data security and ethical research practices.

## Results

### Demographic and clinical characteristics

A total of 1,380 children were enrolled in this study. Table 1 presents the baseline characteristics of the study population. The cohort comprised 844 boys (61.2%) and 536 girls (38.8%), with a mean age of 4.70 ± 0.92 years. The age distribution was relatively uniform across the three age groups (3–4, 4–5 and 5–6 years), demonstrating balanced representation within the preschool-aged study population.

**Table 1:** Baseline demographic and clinical characteristics of preschool-aged children included in the secure multi-party computation respiratory pathogen decision-support study (*N* = 1,380).

Characteristic	n (%) or Mean ± SD
Age (years)	4.70 ± 0.92
Sex	
Male	844 (61.2)
Female	536 (38.8)
Weight (kg)	18.5 ± 6.4
Follow-up duration (months)	3.8 ± 0.6

Abbreviations: N, number of children; SD, standard deviation; SMC, secure multi-party computation.

### Respiratory pathogen detection patterns analysis

Respiratory pathogen screening revealed significant variations in detection rates across different markers. As shown in Table 2, immunoglobulin M (IgM) antibodies against *Mycoplasma pneumoniae*, used as markers of atypical bacterial infection, demonstrated the highest positive detection rate at 41.30%, with 14 positive and 43 weakly positive cases among 138 tests. Influenza viruses showed moderate positivity rates, with influenza B virus IgM at 9.13% and influenza A virus IgM at 7.39%. Immunoglobulin M antibodies against *Chlamydia pneumoniae*, also used as markers of atypical bacterial infection, yielded no positive results across all tests performed.

**Table 2:** Respiratory pathogen immunoglobulin M detection results and positivity rates among preschool-aged children undergoing respiratory pathogen screening.

Pathogen category and marker	Total tests	Positive	Weakly positive	Negative	Positivity rate (%)
Atypical bacteria: Mycoplasma pneumoniae IgM	138	14	43	81	41.30
Virus: Influenza B virus IgM	230	2	19	209	9.13
Virus: Influenza A virus IgM	230	2	15	213	7.39
Virus: Respiratory syncytial virus IgM	138	0	5	133	3.62
Virus: Adenovirus IgM	138	0	3	135	2.17
Virus: Coxsackievirus IgM	138	0	2	136	1.45
Virus: Parainfluenza virus IgM	230	0	1	229	0.43
Atypical bacteria: Chlamydia pneumoniae IgM	138	0	0	138	0.00

Abbreviations: IgM, immunoglobulin M; SMC, secure multi-party computation. Positivity rate was calculated as positive plus weakly positive results divided by total tests.

### Clinical diagnoses and treatment outcomes

The distribution of underlying clinical conditions and their associated descriptive treatment outcomes is presented in Table 3. These outcomes reflect the complete hospital care pathway, including protocolised treatment, clinician review and follow-up, rather than an isolated causal effect of SMC encryption. Children receiving malignancy-related chemotherapy represented the largest underlying-condition category, accounting for 289 children (20.91%). Among these children, 65.4% showed improvement and 31.8% achieved complete cure. Children with immune thrombocytopenia, the second most common condition, demonstrated favourable descriptive outcomes, with an improvement rate of 88.4%.

**Table 3:** Underlying clinical conditions and descriptive treatment outcomes among preschool-aged children in the secure multi-party computation respiratory pathogen decision-support study.

Underlying condition/acute clinical context	Total cases	Improved	Cured	Unchanged	Other
Malignancy-related chemotherapy	289	189 (65.4)	92 (31.8)	5 (1.7)	3 (1.1)
Immune thrombocytopenia	95	84 (88.4)	9 (9.5)	1 (1.1)	1 (1.0)
Kawasaki disease	69	12 (17.4)	57 (82.6)	0 (0.0)	0 (0.0)
Sepsis	57	42 (73.7)	13 (22.8)	1 (1.8)	1 (1.7)
Aplastic anemia	54	48 (88.9)	5 (9.3)	0 (0.0)	1 (1.8)
Others	816	568 (69.6)	237 (29.0)	1 (0.1)	10 (1.3)

Abbreviations: SMC, secure multi-party computation. Other denotes documented outcomes outside the improved, cured or unchanged categories.

### Secure multi-party computation system performance

Table 4 presents the technical performance and reference-standard concordance comparison between the SMC system and the non-encrypted computational comparator. The SMC system achieved diagnostic-support concordance of 94.7% compared with 95.2% in the non-encrypted comparator (*p* = 0.42), demonstrating that the encryption protocols did not significantly compromise the outputs of the same rule-based algorithm. The encrypted model achieved a macro-AUROC of 0.962, precision of 94.0%, recall of 95.1%, F1-score of 94.5% and specificity of 96.8% against the composite clinical reference standard. Processing time increased from 0.15 ± 0.03 s in the comparator to 2.3 ± 0.5 s with SMC (*p* < 0.001). Although this increase was statistically significant, it remained clinically feasible for non-emergency diagnostic support because it occurred within the timeframe of routine laboratory result review. In terms of system reliability and security monitoring, the system achieved 99.97% uptime, with the remaining 0.03% downtime (26 min in total) attributed to scheduled maintenance for security updates and key rotation procedures. No cryptographic failures, unauthorised access events, key-share compromises or protocol violations were detected during deployment. The marginal difference in concordance (0.5%) between the encrypted and non-encrypted systems indicates that SMC technology can maintain algorithmic output equivalence while providing data protection; it does not establish superiority over physician-only care because the comparator was not a clinical-care control group.

**Table 4:** Technical performance, reference-standard concordance and monitored security outcomes of the secure multi-party computation system compared with the non-encrypted computational comparator.

Performance indicator	SMC system	Non-encrypted computational comparator	*p*-value
Diagnostic-support concordance with clinical reference standard (%)	94.7 ± 1.2	95.2 ± 1.1	0.42
Processing time (seconds)	2.3 ± 0.5	0.15 ± 0.03	<0.001
False positive rate (%)	3.2 ± 0.4	3.0 ± 0.3	0.38
False negative rate (%)	2.1 ± 0.3	1.8 ± 0.2	0.21
System uptime (%)	99.97	100	N/A
Detected cryptographic failures or unauthorised access events (n)	0 detected	N/A^a^	N/A
Physician concordance (%)	89.3 ± 2.1	90.1 ± 1.9	0.51
Treatment prediction accuracy (%)	87.6	N/A	N/A
AUROC	0.962	0.965	0.58
Precision (%)	94.0	94.4	0.49
Recall/Sensitivity (%)	95.1	95.6	0.46
F1-score (%)	94.5	95.0	0.47
Specificity (%)	96.8	97.0	0.38
Multiplicative depth	2	N/A	N/A
Clinical feasibility threshold	<5 s target met	<5 s target met	N/A

a The computational comparator used the same 1,380 children's records and identical clinical algorithm without SMC encryption; it was used to evaluate encryption-related overhead and output equivalence, not superiority over clinician care. Diagnostic-support concordance and related classification metrics were calculated against the composite clinical reference standard described in the Methods. Treatment-outcome rows are descriptive clinical outcomes from the care pathway and are not evidence that encryption caused recovery. The security-monitoring row reports detected events during the monitored deployment period and should not be interpreted as proof of absence of long-term adversarial risk. Abbreviations: AUROC, area under the receiver operating characteristic curve; F1-score, harmonic mean of precision and recall; N/A, not applicable; SMC, secure multi-party computation.

### Age-specific treatment outcomes

Treatment outcomes showed consistency across different age groups, as illustrated in Table 5. Improvement rates were similar across all age groups, ranging from 68.1% to 68.6%, with cure rates consistently around 30%. This descriptive age pattern should not be interpreted as evidence of age-specific treatment efficacy; it indicates that care-pathway outcomes were broadly similar within the restricted 3–6-year-old study population.

**Table 5:** Descriptive treatment outcomes by age group among preschool-aged children included in the secure multi-party computation respiratory pathogen decision-support study.

Age group (years)	Total cases	Improved (%)	Cured (%)	Unchanged (%)	Other (%)
3–4	458	312 (68.1)	137 (29.9)	4 (0.9)	5 (1.1)
4–5	463	316 (68.3)	138 (29.8)	3 (0.6)	6 (1.3)
5–6	459	315 (68.6)	138 (30.1)	1 (0.2)	5 (1.1)

Abbreviations: SMC, secure multi-party computation. Other denotes documented outcomes outside the improved, cured or unchanged categories.

### Follow-up analysis

Long-term follow-up data were available for 98.3% of children (1,356/1,380). The sustained improvement rate at 3 months was 94.5%, with only 3.2% of cases requiring substantial treatment modification during the follow-up period. The SMC system's predictive accuracy for treatment response remained 87.6% throughout the follow-up period, although these observational clinical outcomes should be interpreted in the context of physician review, protocolised treatment and longitudinal follow-up rather than as direct evidence that encryption improved recovery.

## Discussion

This study presented implementation evidence for SMC technology as a privacy-preserving clinician-reviewed decision-support framework for respiratory pathogen diagnosis and treatment planning in preschool-aged children. The high positive outcome rate of 98.3% observed in this study should be interpreted cautiously because it reflected the combined effects of protocolised care, physician review and follow-up rather than the cryptographic layer alone. The high detection rate of *Mycoplasma pneumoniae* IgM aligned with recent epidemiological studies in children and reinforced the need to classify *M. pneumoniae* as an atypical bacterial pathogen rather than a virus (19, 20).

The significant prevalence of children receiving malignancy-related chemotherapy (20.91%) in our study population highlighted the complex interplay between respiratory infections and immunocompromised states. The 97.2% positive outcome rate in this subgroup was favourable, but it cannot be attributed solely to SMC. The observed outcomes were more appropriately associated with structured treatment protocols, close follow-up and clinician oversight supported by secure multi-parameter analysis, especially because oncology-related datasets require cautious privacy and governance safeguards.

The observed variation in respiratory pathogen detection patterns provided valuable insights into the epidemiological landscape of respiratory infections in children. The relatively high positivity rates for influenza viruses (influenza B virus: 9.13%; influenza A virus: 7.39%) were interpreted as marker-level findings rather than standalone proof of acute infection. Respiratory viral infections can have clinically important outcomes in children who are immunocompromised, and single-point IgM testing has important limitations: IgM may be absent early in infection, persist after a previous infection or be unreliable in children with impaired humoral immunity (21). Therefore, the system was designed to incorporate PCR cycle threshold values and inflammatory biomarkers, including C-reactive protein and procalcitonin, whenever these data were available, while maintaining governance safeguards for sensitive child health data (22).

The system's technical performance metrics, particularly the 94.7% diagnostic-support concordance, macro-AUROC of 0.962 and F1-score of 94.5%, demonstrated that encryption did not materially reduce classification performance compared with the non-encrypted computational comparator. These metrics should be interpreted as agreement with the composite clinical reference standard and output equivalence testing, not as independent proof of clinical superiority of the underlying rule set. The false-positive (3.2%) and false-negative (2.1%) rates were low overall, although interpretation of negative serological results required additional safeguards in children who are immunocompromised. The processing time of 2.3 ± 0.5 seconds reflected the deliberately shallow multiplicative depth of the decision circuit and remained compatible with the predefined non-emergency diagnostic-support workflow.

The consistent descriptive treatment outcomes across different age groups (improvement rates ranging from 68.1% to 68.6%) suggested that care-pathway outcomes were similar within the enrolled 3–6-year-old population. However, the study did not include infants, older children or adolescents, whose immune responses, infection spectra and diagnostic thresholds may differ substantially. Age-specific respiratory infection burden and prevention literature supports caution when extrapolating preschool findings to other age groups of children (23, 24). Accordingly, the algorithmic thresholds and safety rulesets should be interpreted as preschool-specific rather than general defaults for all children.

The management of children with immune thrombocytopenia (88.4% improvement rate) and cases of Kawasaki disease (82.6% cure rate) was descriptively compatible with embedding secure multi-parameter analysis within care for complex inflammatory or immunological contexts, but these outcomes do not prove that SMC caused clinical recovery. This interpretation is consistent with evidence that decision-support systems should complement, rather than replace, clinician diagnostic judgement (25). For children with suspected inborn errors of immunity, especially humoral immune defects, the system incorporated a safety mechanism that prioritised PCR or other molecular testing and prevented a negative IgM result from being interpreted as excluding infection. This safeguard is essential because laboratory detection thresholds and result interpretation can affect infectious disease classification, especially when serology-based models are applied to children unable to mount an adequate antibody response (26).

The system's 99.97% uptime reflects real-world clinical deployment constraints, with the remaining 0.03% planned downtime necessary for critical security maintenance, including key rotation, security patch implementation and audit-log review. This scheduled maintenance approach supported security integrity while minimising clinical disruption. The absence of detected cryptographic failures or unauthorised access events should be interpreted within the study's monitored deployment model. A monitored security event was defined as any instance in which unauthorised parties gained access to decrypted patient data, the private key or key shares were compromised, the multi-party computation protocol was violated or audit logs showed anomalous access patterns. The monitoring system performed real-time analysis of computation requests, verified protocol compliance and maintained cryptographic proofs of computation integrity.

Although no cryptographic failures or unauthorised access events were detected during the study period, the closed and carefully monitored clinical deployment did not simulate persistent external adversaries, insider collusion or long-term attack evolution. No system can guarantee absolute security indefinitely; therefore, continuous monitoring, independent penetration testing, regular security updates and key-governance audits remain necessary for broader implementation. The minimal accuracy difference (0.5%) between the encrypted and non-encrypted systems demonstrates that privacy-preserving computation need not compromise algorithmic output. The increased processing time (2.3 vs. 0.15 s) was statistically significant but remained clinically acceptable for non-emergency diagnostic support on the CPU-based hospital private-cloud deployment described in the Methods. Real-world implementation also requires attention to computational and communication cost, hardware maintenance, network latency, key-management governance, staff training and the learning curve for non-technical clinicians (27–29).

The recent literature supports cautious integration of AI and secure computation into diagnostic pathways for children. Large language model studies in paediatric diagnosis suggest that AI can complement clinician judgement but still requires clinical oversight (30). Contemporary reviews of paediatric bacterial pneumonia emphasise multimodal data integration for distinguishing bacterial, atypical bacterial and viral disease (31). Martinson et al. (32) reported current AI/ML approaches for inborn errors of immunity, supporting the need for algorithms to recognise immune-defect phenotypes rather than relying solely on antibody responses. Ballhausen et al. (33) described privacy-friendly evaluation of patient data with secure multiparty computation in a European pilot study, providing a relevant clinical benchmark for SMC feasibility, latency and governance. Epidemiological studies in China have also shown substantial *M. pneumoniae* activity in children, supporting the inclusion of atypical bacterial pathways and local resistance-aware treatment logic (34, 35).

Several limitations should be acknowledged. The observational design and absence of a physician-only clinical control group mean that the study evaluates SMC feasibility, reference-standard concordance and encrypted-vs.-non-encrypted output equivalence rather than clinical superiority. The composite reference standard relied on routine clinical documentation and standard-of-care laboratory evidence, so misclassification remains possible when PCR or molecular testing was unavailable. The weighted rules were guideline-derived and locally parameterised rather than externally trained across multiple centres, which may limit portability to hospitals with different pathogen epidemiology, antimicrobial resistance patterns, laboratory assays or workflow constraints. Security monitoring was performed in a controlled deployment and did not reproduce persistent adversarial attacks or collusion between parties. Finally, the cohort was restricted to children aged 3–6 years, making dedicated validation necessary before application to infants, older children or adolescents.

## Conclusions

This study demonstrated that SMC technology provided a feasible framework for auxiliary respiratory pathogen diagnosis and treatment planning in preschool-aged children at a single tertiary centre. The system's performance across complex clinical scenarios, including children who were immunocompromised, supported its potential value as clinician-reviewed decision support within the validated 3–6-year age range. The combination of high reference-standard diagnostic-support concordance, strong monitored security measures and clinically feasible processing time suggested that SMC may be useful in respiratory infection workflows for preschool-aged children. However, the current algorithms, decision thresholds and safety rulesets were validated only for preschool-aged children, and extrapolation to infants, older children or adolescents requires dedicated age-specific validation across broader pathogen spectra, immune profiles and clinical protocols.

## Data Availability

The original contributions presented in the study are included in the article/Supplementary Material, further inquiries can be directed to the corresponding author.
